# Space-Focused Stereotypes About People Living With HIV/AIDS and the Effects on Community-Approaching Willingness

**DOI:** 10.3389/fpsyg.2022.772639

**Published:** 2022-04-15

**Authors:** Fangfang Wen, Yang Wang, Bin Zuo, Jian Yang, Yalan Qiao, Hanxue Ye, Zengqi Luo

**Affiliations:** School of Psychology, Central China Normal University, Wuhan, China

**Keywords:** space-focused stereotype, people living with HIV/AIDS (PLWHA), stigmatized group, community-approaching willingness, mediation effect, geographical segregation

## Abstract

Targeting people living with Human Immunodeficiency Virus (HIV), this research examined the prevalence of space-focused stereotypes and their underlying mechanism on behavioral inclinations. Study 1 adopted the explicit nomination and implicit Go/No-Go association tests to explore the existence of space-focused stereotypes of people living with HIV/AIDS. The results demonstrated that space-focused stereotypes were only manifested explicitly with characteristics such as messy, dirty, and gloomy. Study 2 demonstrated a more negative evaluation and community-approaching willingness for communities that include people living with HIV/AIDS than those without HIV/AIDS. Additionally, space-focused stereotypes were found to have an indirect influence on community-approaching willingness; the influence was mediated by both emotional (threat perception) and cognitive factors (community evaluation). These results indicate the deviation of explicit and implicit space-focused stereotypes. More importantly, it revealed that space-focused stereotypes decreased community evaluation and influenced behavioral inclination. This research suggested the existence of space-focused stereotypes on another stigmatized social group. Characteristics of space (e.g., geographical segregation) might be the key to forming space-focused stereotypes.

## Introduction

In contrast to the halo effect, which suggests a positive impression of others with only the knowledge of one positive trait, there exists a “jinx effect,” which suggests the negative impression of others with only the knowledge of one negative trait. Previous literature has described the space-focused stereotype and demonstrated how people extend their negative stereotypes toward other social groups into their physical space (Bonam et al., 2016). So far, previous studies on space-focused stereotypes have mainly examined stereotypes related to Black Americans (Bonam et al., 2016, 2018). For Black Americans, geographical segregation exists between their and White Americans’ living area in the US. However, considering the history of racial segregation in the US, there are complex historical-cultural factors underlying the geographical segregation of Black Americans (Grigoryeva and Ruef, 2015). Thus, previous findings related to space-focused stereotypes could be cultural-specific (Bonam et al., 2017) and needed to be addressed in other social groups with the nature of geographical segregations. In China, people living with HIV/AIDS (PLWHA) are represented geographically segregated social groups (Qin, 2017). Moreover, similar to Black Americans in the US (Yantis and Bonam, 2021), it is also a stigmatized group perceived as having lower socio-economic status (Li et al., 2008). Therefore, could the space-focused stereotypes also be found in other social groups (e.g., PLWHA) with geographical segregation? Exploring the space-focused stereotypes in PLWHA helped reveal the connection between geographical segregation and space-focused stereotypes more directly.

Stereotypes are a fixed idea or schema toward a specific social group (Fiske, 2004). Numerous researchers in this area focus on how context, including physical and social contexts, influences social cognition (Fiske et al., 1998). Researchers have found an interrelation between physical context and stereotypes (Oishi, 2014; Meagher, 2020); that is, physical context influences the content of stereotypes (Wittenbrink et al., 2001; Trawalter et al., 2012; Motyl et al., 2014), while stereotypes also influence the evaluation of physical objects (Meagher, 2017).

Based on these findings, Bonam et al. (2016) extended the influence of racial stereotypes into physical space and found that negative evaluations of Black Americans’ living space further decreased their environmental protection willingness. Their further research found the space-person asymmetry in racial stereotype content and application. That is, stereotypes regarding Black and White Americans’ living spaces were stronger than Black and White American people, and space-focused rather than person-focused racial stereotype application is more prominent within Americans (Bonam et al., 2018). Therefore, researchers proposed that space-focused stereotypes are a manifestation of racial stereotypes in space (Bonam et al., 2016, 2018).

Previous studies and findings related to space-focused stereotypes were exclusively based on Black Americans. It is worth noting that the racial segregation between “Black” and “White” has a long history that related to prejudice and bias against Black Americans (Grigoryeva and Ruef, 2015). Thus, the findings of prior work might limit to the US/Western culture (Bonam et al., 2017). As Bonam et al. (2018) have revealed the importance of space characteristics in space-focused stereotypes, we inferred that the existing geographical segregation might be the key to the formation of the space-focused stereotypes, even in other social groups. In addition, methods used in prior research mainly relied on explicit self-report (Bonam et al., 2016, 2018), which could be interference by social desirability (Payne and Hannay, 2021). There is lacking exploration of space-focused stereotypes in implicit measures.

Our present research tried to address the above-mentioned problems in China. Consistent with the geographical segregation characteristics of Black Americans’ living space, this research selected another geographically segregated social group in China, the PLWHA. It was well-established that PLWHA tends to cluster in their living areas worldwide (Zulu et al., 2014; Jeefoo, 2016; Chávez, 2021). This pattern is also true in China as the earlier transmission process of HIV (Qin, 2017). In the 1980s, people in villages tried to increase their salary by donating blood, and many were infected due to plasma-collection contamination (Zhang et al., 2006; Yan et al., 2013; Wu et al., 2019). Accordingly, geographical segregation became one of the prominent characteristics of PLWHA. For example, using epidemiological analyses, a study examined the regional differences of HIV/AIDS prevalence in China from 2004 to 2016 and found a significant geographical distribution pattern of HIV/AIDS prevalence in China (Qiao et al., 2019). Moreover, PLWHA is also likely to contact and live with their ingroup (Horvath et al., 2012) and thus create geographical segregation from the majority social groups.

Therefore, the first aim of the present research was to explore people’s space-focused stereotypes of PLWHA in China and its influence on people’s behavioral inclination. Both explicit and implicit measurements were deployed to amend the deficiency of self-report in a previous study (Bonam et al., 2018). Comparing the characteristics of Black Americans and PLWHA, we predicted a significant negative space-focused stereotype for PLWHA in China, and people tend to avoid contacting the living space of PLWHA for the following reasons.

Black Americans and PLWHA are typical stigmatized social groups in the US and China, respectively (Zhang, 2011; Li et al., 2017; Yantis and Bonam, 2021). Stigma refers to demeaning and insulting labels society imposes on a specific individual or group (Guan, 2007; Zhang and Yu, 2007). As the transmission of HIV is generally connected to specific behaviors such as drug addiction and unprotected sexual behavior, people tend to condemn PLWHA as being morally deficient (Mak et al., 2006; Zhang et al., 2016). In China, although the government announced policies to support PLWHA since 2006 (Wu et al., 2019), biases and prejudices are inevitable once others know an HIV-positive case. For example, PLWHA are more likely to lose their jobs, be rejected when applying to rent a house and become isolated from friends and family (Link and Phelan, 2006; Li et al., 2008; Yang et al., 2021). It is worth noting that PLWHA is different from Black Americans as HIV infections could be hidden from others (concealable stigma; Quinn and Earnshaw, 2013). In this case, although the general public could not identify PLWHA through their appearance, the fact that they are from “AIDS villages” might result in the avoidance inclination within perceivers (Baunach and Burgess, 2013). However, from the perspective of PLWHA themselves, it is a matter of responsible behavior to tell their partners about their infections to prevent the transmission of HIV. Examining the public’s negative stereotypes and avoidance behavior is crucial before PLWHA can accept the fact that they are infected.

The second aim of this study was to reveal the mechanism underlying avoidance behaviors toward PLWHA’s living space. Consistent with previous literature (Bonam et al., 2016), this research utilized community-approaching willingness to measure avoidance of PLWHA and further investigate its relationship with space-focused stereotypes. Specifically, we used community-approaching willingness to assess participants’ willingness to move their house near a target community. In their investigations of mechanisms underlying a particular behavior, prior researchers have always separated emotion and cognition as distinct pathways (Akinola and Mendes, 2008; Zuo et al., 2019). Therefore, we also addressed potential emotional and cognitive factors as mediators in the relationship between space-focused stereotypes and community-approaching willingness.

As a systematic bias of social cognition, stereotyping is closely related to cognitive factors (Zuo et al., 2006) that evoke emotional reactions and prejudiced behaviors (Cuddy et al., 2008). The emotional reactions for PLWHA have been well evidenced in previous research. For example, people demonstrated negative emotions toward PLWHA and were afraid of getting infected and, subsequently, avoided contact with PLWHA (Berger et al., 2001; Popova, 2007; Pickles et al., 2009; Polonsky et al., 2015). Thus, the threat of HIV infection could be the reason for negative attitudes toward PLWHA. For the present research, threat perception was predicted to mediate the relationship between space-focused stereotypes and behavioral inclinations; feelings of threat, fear, and anxiety were indicators of threat perception (Zheng and Zhao, 2016).

Regarding cognitive reactions, the evaluation of the community environment was the critical cognitive factor in assessing the living space of a stigma social group (Bonam et al., 2016). Similar to Black Americans, PLWHA is also perceived as having a lower socio-economic status (Link and Phelan, 2006; Li et al., 2008; Yang et al., 2021). Therefore, this research also measured community evaluation through items related to the general socio-economic conditions. We assumed that community evaluation might significantly mediate the influence of space-focused stereotypes on community-approaching willingness.

In conclusion, this study has two aims. First, we examined the space-focused stereotypes in evaluations of PLWHA by focusing on both explicit (Study 1A) and implicit stereotypes (Study 1B). Second, this study investigated the influence of space-focused stereotypes on behavioral inclinations and furthered the possible mediation effects of emotional and cognitive factors (Study 2).

## Study 1: Space-Focused Stereotypes of PLWHA

Study 1 aimed to explicitly and implicitly test space-focused stereotypes of PLWHA. Study 1A used a self-reported method to investigate explicit space-focused stereotypes of PLWHA, and Study 1B used the Go/No-Go association test (GNAT) to measure implicit space-focused stereotypes of PLWHA.

### Study 1A: Explicit Measures of Space-Focused Stereotypes

#### Participants

A total of 87 students (*M*_*age*_ = 19.72, *SD* = 0.96) were recruited from a university in Wuhan, China, including 19 males and 68 females. A sensitivity analysis revealed that, at a critical alpha of 0.05, the sample size (87) had a conventional 80% power to detect small effects of *d* = 0.27 in a one-sample *t-*test. All participants volunteered to participate in the study and provided informed consent before the formal testing. None of the participants were excluded because of the validity of their responses.

#### Procedures and Materials

The data was collected through an online survey platform^1^ in China. This study was carried out following the recommendations of the American Psychological Association (APA) ethical guidelines, and the protocol was approved by the Ethics Committee of Central China Normal University. All participants provided informed consent before the formal experiment was conducted. The informed consent form included a brief description of our study, the confidentiality of their data (i.e., regarding remaining anonymous in any publication related to this study), and their rights to withdraw from the experiment at any time. Researchers’ contact information was also provided so that participants could inquire about any further details of the study. After reading the consent form, the participants indicated their willingness by checking the “I agree” option. The informed consent procedure was identical for all the following studies.

In the formal testing, the participants completed a questionnaire consisting of a free association test and a space-focused stereotype measurement after they had submitted the consent forms.

The free association test asked participants to generate at least five characteristics describing PLWHA’s living space in China. Then, the participants were asked to evaluate the valence of each character they generated, from −3 (very negative) to 3 (very positive). Additionally, the participants also evaluated the extent to which the public agrees to each characteristic on an 11-point Likert scale (0 = strongly disapprove, 10 = strongly approve). The evaluated consensus of the public also indicates the participants’ confidence in each character.

The measurements of space-focused stereotypes of PLWHA were revised from Bonam et al. (2016), including three positive and four negative items related to living spaces. This approach was applied in the present research because, similar to some other groups, such as Black Americas, PLWHA are also being perceived as economically disadvantaged (Li et al., 2008; Cui et al., 2017; Yang et al., 2021), and previous studies of space-focused stereotypes have also included items related to socioeconomic status. The three positive items we used were: *great access to banks or savings and loan institutions*, *well-kept houses and properties*, and *great neighborhood shopping*. The four negative items were: *poor neighborhood safety*, *poor city services* (e.g., street cleaning or garbage collection services), *industrial facilities nearby* (e.g., power plants and incinerators), and *low-quality public schools*. All the items were translated and back-translated from English to Chinese by two individuals fluent in both languages. The participants were asked to rate items about the living area of a person with HIV-AIDS on a 10-point Likert scale (1 = highly inconsistent, 10 = highly consistent). The Cronbach’s α of the scale was 0.79, which suggested the reliability of this result.

#### Coding

Three coders who did not know the study’s purpose were asked to encode the participants’ generated characteristics of PLWHA’s living space. First, the coders had to distinguish the features describing spaces from those describing the PLWHA themselves. Then, the coders had to encode the characteristics that described the living space into different categories according to their meanings. Finally, the coders checked their coding categories with each other. For inconsistent coding, the coders discussed them until they reached a consensus.

#### Results of Coding

All the collected characteristics were originally written in Chinese and translated into English in the main text. The participants nominated a total of 697 words that described the characteristics of PLWHA’s living space; 632 validated characteristics remained after the coding procedure. Excluded characteristics were those that described the personality of PLWHA and their feelings about PLWHA. The classified characteristics accounted for 90.7% of all characteristics. The coding procedure generated 13 categories of negative space descriptions (452 negative characteristics) and seven categories of positive space descriptions (121 positive characteristics). Six categories (59 characteristics) were categorized as neutral. The top ten categories ranked by the number of characteristics are listed in Table 1 (see Supplementary Table 1 in Supplementary Materials for all categories, and Supplementary Table 3 for categories in Chinese). The sum of characteristics in the top ten categories accounted for 66.9% of the validated characteristics.

**TABLE 1:** Coded categories of space-focused stereotypes on PLWHA and their corresponding valences and evaluated public consensus.

Category	Examples	*n* ^a^	Valence			Consensus		
			*M* (*SD*)	*T*	Cohen’s *d*	*M* (*SD*)	*T*	Cohen’s *d*
Mess	chaos, disorder	62	−1.73(1.30)	−10.49***	–1.33	5.66 (2.23)	2.33*	0.30
Dirty	unsanitary, dirty	57	−2.14(1.26)	−12.83***	–1.70	6.09 (2.39)	3.43**	0.45
Dark	dark, gloomy	54	−1.44(1.62)	−6.55***	–0.89	4.98 (2.19)	–0.06	–0.01
Narrow	narrow, small	52	−1.19(1.07)	−8.06***	–1.12	5.46 (1.91)	1.74	0.24
Chilly	white, empty	39	−0.87(0.89)	−6.09***	–0.95	5.53 (1.84)	1.76	0.29
Isolation	isolated, quarantined	37	−1.84(1.14)	−9.78***	–1.63	6.50 (2.11)	4.37***	0.71
Clean	clean, germfree	35	1.77 (1.09)	9.64***	1.63	5.40 (2.32)	1.02	0.17
Remote	secluded, remote	30	−0.90(1.40)	−3.53***	–0.64	6.50 (2.03)	4.05***	0.74
Dilapidated	poverty, shabby	29	−1.52(1.24)	−6.58***	–1.22	6.03 (1.66)	3.36**	0.62
Neat	organized, orderly	28	1.79 (1.32)	7.18***	1.36	4.96 (1.77)	–0.11	–0.02

*^a^The total number of characters generated by participants.*

***p < 0.001, **p < 0.01, *p < 0.05.

*The characteristics and categories listed in this table were translated from Chinese. Please see Supplementary Table 3 in Supplementary Materials for their initial Chinese version.*

#### Valence and Consensus of the Characteristics

The valence and evaluated consensus of the coded categories were analyzed using SPSS 27.0. Each participant averaged the valence of the characteristics they generated, thus creating their attitudes toward PLWHA’s living space. Most participants (71.52%) perceived the living space of PLWHA as negative (statistical significantly lower than 0), *χ^2^* (1, *N* = 87) = 5.16, *p* = 0.02, Φ = 0.06.

Then, the valence was averaged across all validated characteristics; it was found that the participants had negative space-focused stereotypes of PLWHA overall, *M* = −0.73, *SD* = 1.83, *t*(610) = −9.89, *p* < 0.001, Cohen’s *d* = −0.40. By calculating the mean valence of the top ten categories, the results demonstrated that except for the positive categories of clean and tidy (see Table 1), other categories were all negative (*p*_*s*_ < 0.01). Additionally, there was a significant negative correlation between the number of characteristics within each category and its valence, *r*(26) = −0.46, *p* = 0.017; that is, the negative categories were more frequently mentioned by the participants.

For the consensus evaluation, a higher rating indicated the perceived representation of each character. The consensus rating was higher than the midpoint (5), *M* = 5.63, *SD* = 2.10, *t*(610) = 7.38, *p* < 0.001, Cohen’s *d* = 0.30, indicating confidence on their self-generated characteristics. Moreover, there was a significant difference in the consensus between the negative and positive categories, *F*(1,610) = 8.43, *p* = 0.004, *η^2^* = 0.014. Further analysis demonstrated that the participants perceived the negative categories as more representative, *M* = 5.77, *SD* = 2.09, compared with positive categories, *M* = 5.21, *SD* = 2.09.

#### Space-Focused Stereotypes of PLWHA

Using the scale developed by Bonam et al. (2016), the result of one sample *t*-test (compared to 5.5) demonstrated negative space-focused stereotypes of PLWHA. The stereotyping was especially illustrated in the evaluation of public safety, city services, school quality, financial institutions, and houses and property conditions (Table 2).

**TABLE 2:** Explicit measurement of space-focused stereotypes on PLWHA.

Items	*M*	*SD*	*T*	Cohen’s *d*
Poor neighborhood safety	6.29	2.20	3.33**	0.36
Poor city services (e.g., street cleaning or garbage collection)	6.23	2.17	3.14**	0.34
Industrial facilities nearby (e.g., power plants and incinerators)	5.31	2.00	–0.88	–0.10
Low quality public schools	6.23	2.18	3.12**	0.34
Great access to banks or savings and loan institutions	5.01	2.02	−0.26*	–0.24
well-kept houses and properties	4.36	1.98	−5.38***	–0.58
Great neighborhood shopping	5.15	2.15	–1.52	–0.16
All items ^a^	6.08	1.41	3.83***	0.41

***p < 0.001, **p < 0.01, *p < 0.05.

*^a^All items indicate the averaged rating on above seven items, the ratings on positive items were reversed so that a higher rating means a more negative space-focused stereotype.*

### Study 1B: Implicit Measurement of Space-Focused Stereotypes

#### Participants

The G*power 3.1 was used to calculate the sample size for this experiment; it demonstrated that for the medium level of effect size (Cohen’s *d* = 0.45-0.55) with 0.05 significant level (two tails), 28 to 41 participants were needed to detect the power of 0.8 in a paired-sample *t*-test. Therefore, a total of 43 participants were recruited (14 males and 29 females, *M_*age*_* = 19.79, *SD* = 2.86). As the implicit measurement was a single-category-GNAT, participants’ responses were preliminarily analyzed to select qualified data from the sample (Nosek and Banaji, 2001). The procedures of the preliminary analysis were as follows: (a) The rate of hit and false alarm of each participant was calculated and then converted into *z-scores*; (b) The sensitivity *d’* score for the single category was calculated by subtracting the hit rate from the rate of false alarm; finally, (c) Participants with a *d’* score less than zero were eliminated, which meant they were not fully involved in the experiment. According to the preliminary analysis, two males were excluded, so the final sample size was 41 (12 males and 29 females, *M_*age*_* = 19.88, *SD* = 1.90). For the rest of the participants, their *d’* scores were higher than 0 (*M* = 19.88, *SD* = 1.90), and their average accuracy was 83.69%.

#### Materials

GNAT is a traditional paradigm used to measure implicit attitudes or beliefs. It reflects potential attitudes toward categories through the difference in reaction time and accuracy between congruent pairings (category and attributes that are stereotypically consistent) and incongruent pairings (category and attributes that are stereotypically inconsistent). GNAT was suitable for this study because it can measure implicit attitudes toward a single category (Nosek and Banaji, 2001; Wen and Zuo, 2007), in this case, the implicit space-focused stereotypes of PLWHA.

Four kinds of words must be provided in GNAT, including synonyms of target category, control category, positive attributes, and negative attributes (see Supplementary Table 2 in Supplementary Materials for details about attributes). The synonyms of PLWHA and the control category were self-generated. The general public were regarded as the control category based on previous research (Yang et al., 2011). As the present study focused on the implicit space-focused stereotypes, the attributes were positive and negative characteristics generated from Study 1A. Therefore, 10 negative and 10 positive characteristics were selected from the results of the free association test in Study 1A. We recruited another 72 participants (13 males and 59 females, *M* = 25.21, *SD* = 12.12) to evaluate the valence of these characteristics on a 7-point Likert scale (1 = very negative, 7 = very positive). The eight most positive and negative characteristics were selected as the attributes used in Study 1B (see Supplementary Table 2 in Supplementary Materials). The participants’ ratings of positive and negative attributes were averaged respectively to compare their differences. The results revealed a significant difference between the positive (*M* = 5.74, *SD* = 0.83) and negative attributes (*M* = 2.59, *SD* = 0.73) on valence ratings, *t*(71) = 19.67, *p* < 0.001, Cohen’s *d* = 2.31. The four unselected characteristics were used as attributes in the practice trials of GNAT.

#### Procedures

The procedure of the single-category GNAT is illustrated in Table 3. The GNAT allowed us to examine the connection between PLWHA and spaces describing positive or negative attributes.

**TABLE 3:** The procedure of single-category GNAT.

Phase	Reaction targets	Number of trials	Trial ratio of Go:No-Go
Step 1	HIV category words	16	1:1
Step 2	Positive attributive words	16	1:1
Step 3	Negative attributive words	16	1:1
Step 4	HIV category words & Positive attributive words	32	1:1
Step 5	HIV category words & Negative attributive words	32	1:1

The instructions were presented first, followed by the practice trials. The practice trials were conducted several times until the participants understood the experimental procedure. A slide presenting the target category or attribute was illustrated at the beginning of each block, indicating that participants should provide the “go” responses when they see the trials related to the target category or attribute. The feedback of accuracy and response time were only provided in the practice phase. In the formal experiment, the target category or attribute appeared in the upper left or upper right corner of the screen, while the words that needed to be judged were presented in the middle of the screen. The participants were asked to give their “go” or “no-go” responses once they saw the words presented in the middle of the screen. Each word was presented for a maximum of 600 ms, which met the 500–850 ms standard suggested by Nosek and Banaji (2001).

#### Results

Sensitivity (*d’* score) reflects the ability to distinguish signals from background noise (Nosek and Banaji, 2001). In the GNAT test, the difference in *d’* scores between congruent (target category with negative attributes) and incongruent pairings (target category with positive attributes) indicates whether the connection between congruent pairings is significantly stronger than incongruent pairings. Therefore, a larger difference represents a higher level of stereotypicality.

The results of the present study demonstrated that there was no significant difference in *d’* scores between congruent (*M* = 0.79, *SD* = 0.92) and incongruent pairings (*M* = 0.86, *SD* = 0.93), *t*(40) = 0.31, *p* = 0.76, Cohen’s *d* = 0.08. That is, there were no implicit space-focused stereotypes of PLWHA.

### Discussion

Study 1A found that the participants have explicit negative space-focused stereotypes of PLWHA. Specifically, participants nominated more negative characteristics and categories than positive characteristics and categories, and negative categories were believed to have a higher consensus among the public. Study 1A also measured the space-focused stereotypes utilizing the scale developed by Bonam et al. (2016). The results also demonstrated the existence of negative explicit space-focused stereotypes of PLWHA. However, the results of Study 1B suggested an insignificant implicit space-focused stereotype of PLWHA. These results revealed negative impressions of not only PLWHA traits, behaviors, and appearances but also their living spaces. Moreover, it is mainly manifested in the explicit rather than the implicit level. The specific reasons for the difference between implicit and explicit space-focused stereotypes will be further discussed in the “General Discussion” section.

## Study 2: The Influence of the Space-Focused Stereotype on Behavioral Inclination

Based on Study 1, Study 2 explored how the space-focused stereotypes affect individuals’ behavioral inclinations regarding living spaces of PLWHA, with the following two goals. First, it aimed to understand evaluations and behavioral inclinations toward the living spaces of PLWHA versus those without HIV/AIDS. Second, it focused on the space-focused stereotype targeting PLWHA and its behavior pathway to verify the dual-path model of emotion and cognition.

### Participants

G*power 3.1 was used to calculate the sample size for this experiment; it demonstrated that for the medium level of effect size (Cohen’s *d* = 0.45-0.55) with a 0.05 significant level (two tails) and an allocation ratio of 2:1, 120 to 178 participants were needed to detect the power of 0.8 in an independent sample *t*-test. Therefore, a total of 120 undergraduates (35 males and 85 females, *M*_*age*_ = 20.08, *SD* = 1.87) were recruited from a university in Wuhan. The participants were randomly divided into a control group (the evaluation target was people living without HIV/AIDS, 40 participants, of whom 13 were males) and an experimental group (the evaluation target was PLWHA). More participants (80 participants, 22 males) were included in the experimental group to meet the sample size for analyzing the mediation effect.

As with Study 1, all participants joined the study voluntarily, the anonymity and confidentiality of answers were declared to them, and informed consent was obtained before the formal experiments. None of the participants were excluded.

### Procedure and Materials

The procedure of this study mainly consisted of two parts, including the measurement of space-focused stereotypes and testing after house-related manipulation. After obtaining informed consent, all the participants were given a questionnaire that tested their space-focused stereotypes of PLWHA. The space-focused stereotypes were measured in the same way as Study 1A, which included seven items rated on an 11-point Likert scale, ranging from 0 to 10 (Bonam et al., 2016). Its Cronbach’s α reliability was 0.78.

Following the ratings for space-focused stereotypes of PLWHA, we changed the information between the control and experiment groups. The participants were required to imagine planning to rent a house near their job after finding formal work. Then, information was provided about the house, and the participants were asked to evaluate this house and its surrounding community in terms of threat perception, community evaluation, and community-approaching willingness. Specifically, the participants were given written information on the house owner: “a person living with HIV-AIDs” in the experiment group and “another employee” in the control group. We also presented information about the house in a table to increase a sense of reality for the participants, including house type (flat), floor (third in a seven-floor building), area (85 m^2^), rooms (one living room, two bedrooms, one kitchen, and one bathroom), and orientation (South). The specific information about the house was consistent across the experiment and control groups. Afterward, the participants needed to rate their threat perception, community evaluation, and community-approaching willingness based on given information about the house.

The measurement of threat perception was based on previous research, which selected the feelings of threat, fear, and anxiety as indicators (Zheng and Zhao, 2016). The participants rated the intensity of their emotions when facing PLWHA on a 5-point Likert scale (Cronbach’s α = 0.88).

The community evaluation was conceptualized as an objective evaluation of the community based on information about the house and house owner. Its items were adapted from the test of space-focused stereotypes using generic language, including *community service*, *house and facility maintenance*, *nearby school quality*, *community safety*, *shopping convenience*, *and bank accessibility* (Cronbach’s α = 0.81). For this measurement, items seem to tap into community socio-economic status perceptions. Previous literature demonstrated the connection between PLWHA and their socio-economic status (e.g., Li et al., 2008; Cui et al., 2017); we speculated that Bonam’s approach could reflect participant’s space-focused stereotypes in a specific way. Comparing the items from the space-focused stereotypes, the generic items from community evaluations increase categorical and abstract thinking (Rhodes et al., 2018) and allow participants to rate the community in a more objective form. A 7-point Likert scale was applied, ranging from 1 (extremely poor) to 7 (extremely good).

The evaluation of their community-approaching willingness consisted of two items (rated on a 5-point Likert scale, 1 = entirely negative, 5 = entirely positive). One required the participants to evaluate their willingness to move to the community where the house was located, and the other required them to evaluate their satisfaction with the community if they lived in it (Cronbach’s α = 0.85). Finally, we collected the participants’ demographical information, including age and gender.

### Results

#### Influence of House Owner Type on Community Evaluation

The independent sample *t*-test was used to analyze the difference between the experimental and control groups in community evaluation and community-approaching willingness. It was found that the community evaluation of the control group (*M* = 4.96, *SD* = 0.97) was significantly higher than that of the experimental group (*M* = 4.13, *SD* = 0.70), *t*(118) = 5.38, *p* < 0.001, Cohen’s *d* = 1.03; the community-approaching willingness of the control group (*M* = 3.51, *SD* = 0.80) was also significantly higher than that of the experimental group (*M* = 2.38, *SD* = 0.87), *t*(118) = 6.92, *p* < 0.001, Cohen’s *d* = 1.33. These results indicated a devalued attitude when evaluating spaces related to PLWHA compared to those without HIV/AIDS, and space-focused stereotypes could be the reason for this tendency.

#### Mediation Analysis of Emotional and Cognitive Factors

**FIGURE 1:**
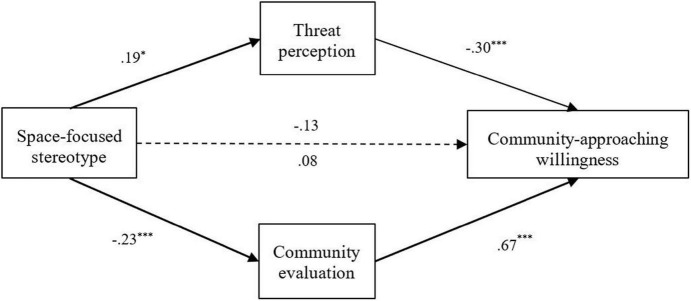
The mediation of threat perception and community evaluation between space-focused stereotype and community-approaching willingness.

The descriptive and correlation results of the variables involved in this study are illustrated in Table 4. Although the negative correlation between the space-focused stereotype and community-approaching willingness was not significant, there were significant correlations between the pathways through threat perception and community evaluation. The planned test for the mediation effect was run using the bias-corrected bootstrap method in PROCESS model 4 (Hayes, 2022), and the coefficients between the variables are illustrated in Figure 1.

**TABLE 4:** The descriptive statistics of the space-focused stereotype, threat perception, community evaluation, and community-approaching willingness.

Variables	*M*	*SD*	1	2	3
Space-focused stereotype	6.00	1.21			
Threat perception	3.12	0.95	0.244*		
Community evaluation	4.12	0.70	−0.393***	–0.039	
Community-approach willingness	2.38	0.87	–0.179	−0.318**	0.514***

It was found that the direct path from the space-focused stereotype of PLWHA to the community-approaching willingness was not significant, *R*^2^ = 0.03, *F*(1,78) = 2.60, *p* = 0.111. However, when threat perception and community evaluation were included in the model, the overall interpretation of this model was significantly improved, *R*^2^ = 0.36, *F*(3,76) = 14.44, *p* < 0.001.

A further analysis revealed the mediation effect of threat perception (−0.08) was statistically significant, bootstrap 95% *CI* = [−0.187, −0.008]. Individuals’ space-focused stereotypes of PLWHA positively predicted their threat perception, β = 0.19, SE = 0.08, *t*(78) = 2.22, *p* < 0.05, which led to a decrease in the willingness to approach their related communities, β = −0.30, *SE* = 0.09, *t*(78) = −3.43, *p* = 0.001. Additionally, the mediation effect of community evaluation was also negatively significant (−0.21), bootstrap 95% *CI* = [−0.370, −0.086]. That is, the higher the space-focused stereotype, the lower the overall evaluation of community, β = −0.23, *SE* = 0.06, *t*(78) = −3.77, *p* < 0.001, which further reduces individuals’ willingness to approach the community, β = 0.67, *SE* = 0.12, *t*(78) = 5.47, *p* < 0.001.

Under the influence of threat perception and community evaluation, the coefficient from the space-focused stereotype to community-approaching willingness changed from negatively insignificant, β = −0.13, *SE* = 0.08, *t*(78) = −1.61, *p* = 0.111, to positively insignificant, β = 0.08, *SE* = 0.07, *t*(78) = 1.11, *p* = 0.207. That is, space-focused stereotypes influenced community-approaching willingness of PLWHA’s living space, and this process was mediated by a cognitive factor (community evaluation) and an emotional factor (threat perception).

### Discussion

In Study 2, by comparing the community-approaching willingness and the community evaluation of the experimental and control groups, it was found that compared to those without HIV/AIDS, individuals’ evaluation of the living space of PLWHA was generally more negative, which was reflected in two aspects: the lower community-approaching willingness and the lower community evaluation. The results also revealed that both threat perception and community evaluation negatively mediated the influence of the space-focused stereotype on individuals’ behavioral inclinations. The space-focused stereotype positively predicted individuals’ threat perception of PLWHA, thus reducing their willingness to approach the communities. Meanwhile, the space-focused stereotype also negatively predicted individuals’ evaluation of PLWHA-related communities, thus reducing their willingness to approach the communities. The results validated the emotional and cognitive dual-path model that space-focused stereotypes affect behavioral inclinations, indicating that space-focused stereotypes of PLWHA would elicit a negative emotional reaction and a negative evaluation of a wider range of physical spaces, leading to avoidance of behaviors.

## General Discussion

This study explored the existence of space-focused stereotypes of PLWHA and the emotional and cognitive mechanisms underlying its behavioral inclinations. Based on the research of Bonam et al. (2016), Study 1A used the free association test and a subsequent survey to explore the content and valence of space-focused stereotypes of PLWHA. The results demonstrated that the participants had negative stereotypes of PLWHA-related spaces, and they would use negative characteristics such as messy, dirty, and gloomy to describe the living space of PLWHA. Additionally, the participants believed that others were more likely to agree with their negative impressions of PLWHA’s living space than positive impressions. These results verified the prevalence of negative space-focused stereotypes of PLWHA.

Negative evaluations of living space for PLWHA are strongly associated with their stigma. As suggested in previous literature, stigma towards PLWHA is prevalent worldwide (e.g., Jeefoo, 2016; Qin, 2017; Chávez, 2021), which is also a focus of medical and psychological literature (Li et al., 2012; Beaulieu et al., 2014; Ren et al., 2014). In China, people tend to attribute HIV infection to unprotected sexual behavior (Cui et al., 2017). Thus, PLWHA faces harsh and severe criticism of their “immorality” than people with other infectious diseases (Mak et al., 2006; Zhang, 2011). Moreover, characteristics nominated in Study 1A, together with the participants’ negative rating on adapted space-focused stereotypes, suggested a lower perception of the socio-economic status of PLWHA, who lose their jobs more frequently and easily, and lack support from others (Link and Phelan, 2006; Li et al., 2008; Yang et al., 2021). It is worth noting that prejudiced perceptions of PLWHA having lower socioeconomic status started to gain popularity in the 1980s (Zhang et al., 2006; Yan et al., 2013; Wu et al., 2019). The negative perception of PLWHA’s economic conditions reflects the persistence of stereotypes and the obstacles encountered by many PLWHA nowadays. Additionally, PLWHA are aware of the possible discrimination and prejudice and the consequence on their economic status (Berger et al., 2001). Therefore, they might conceal their infection of HIV from others, which, in turn, threatened the health of their sexual partners.

Study 1B used a single-category-GNAT paradigm to measure implicit space-focused stereotypes and found that the participants do not have negative perceptions of the living spaces of PLWHA. This result was not expected. However, previous studies exploring the relationship between implicit and explicit stereotypes have demonstrated that they are not always consistent (Rudman and Kilianski, 2000; Kornadt et al., 2016; Matthes and Schmuck, 2017), and environmental factors could cause their differences (Brauer et al., 2000; Smyth and Nosek, 2015). Greenwald and Banaji (1995) proposed that explicit attitude was influenced by recent experience, while implicit attitude was more likely influenced by experiences in early childhood. Cultural and emotional experiences are also linked to the development of implicit attitudes (Rudman, 2004).

For the living space of PLWHA, negative attitudes might derive from newspapers and online media (Nanda and Pramanik, 2010), and so, recent experiences were more likely to be influenced. PLWHA is often associated with prostitution, drug abuse, and other risky behaviors in the public media, leading to negative or stigmatizing views of PLWHA (Zhang and Li, 2005). Additionally, risky behaviors are often associated with low socio-economic status, thus reinforcing explicit negative space-focused stereotypes of PLWHA. However, during early childhood, when implicit stereotypes are formed (Greenwald and Banaji, 1995), children might be less likely to be exposed to the concept of PLWHA. They may not effectively obtain knowledge related to PLWHA from newspapers and online media and thus do not form the implicit space-focused stereotype. Most of the existing childhood studies related to HIV/AIDS have been conducted from the perspective of children infected with HIV (for example, Lee and Rotheram-Borus, 2009), and there is a lack of exploration of children’s impressions and evaluations of others infected with HIV; therefore, this hypothesis needs further testing. Additionally, the focus on implicit space-focused stereotypes was also one of the innovations of the present research. Whether the neutral attitude on the implicit level was a unique characteristic of the space-focused stereotype needs to be addressed in follow-up studies.

In Study 2, we explored the impact of space-focused stereotypes on behavioral inclinations and their mechanisms. The results demonstrated that, compared to evaluations of those without HIV/AIDS, the participants’ evaluations of the living spaces of PLWHA were more negative in terms of community evaluation and community-approaching willingness. Unlike previous research focused on Black Americans (e.g., Bonam et al., 2016; Yantis and Bonam, 2021), this study investigated the space-focused stereotype and its corresponding influence on cognition and behavioral inclinations in another social group with concealable stigma. Although it seems unlikely that people can distinguish PLWHA from non-infected people by appearance, geographical segregation between them does exist worldwide (e.g., Zulu et al., 2014; Jeefoo, 2016; Chávez, 2021), including as in China (Qin, 2017; Qiao et al., 2019; Wu et al., 2019). The results for negative space-focused stereotypes and avoidance behaviors suggest there is a direct relationship between geographical segregation and the cognitive and behavioral response to the segregated space. There seems to be a “mental space” that is generated once a social group is separated from others. Further research could examine this possibility more directly with novel social groups created by the minimal group paradigm. This approach is especially valuable in the background of the COVID-19 pandemic, as quarantine is regarded as an effective measure to control the transmission of the virus.

For PLWHA, geographical segregation and others’ avoidance behavioral inclination are easily detected in their social interactions. In fear of possible social exclusion from others, PLWHA is more likely to conceal their infections, which is irresponsible behavior for their intimate partners and is more likely to result in HIV transmission. Research should continue developing a proper way to intervene the negative evaluation and prejudiced behavior on PLWHA from the public, which is crucial to restrain the prevalence of HIV.

The mediation analysis in Study 2 revealed the mediation effect of threat perception and community evaluation between space-focused stereotypes and community-approaching willingness. For threat perception, higher space-focused stereotypes indicated higher threat perception and further decreased the community-approaching willingness, while higher space-focused stereotypes correlated with a lower community evaluation led to decreased community-approaching willingness. Although the direct relationship between space-focused stereotypes and community-approaching willingness was not significant before and after the inclusion of mediators, the significant mediation effect of threat perception and community evaluation specified the importance of explaining a particular behavior.

The emotional and cognitive pathways are essential mechanisms in explaining behavior. The present research was the first to examine how space-focused stereotypes influence avoidance behavior in China (e.g., Zuo et al., 2019). The factor of community evaluation was considered in Bonam’s initial research (Bonam et al., 2016), while it was regarded as an outcome variable rather than a factor that results in behavioral inclination. Conversely, the emotional response was only examined in evaluating the public’s feelings of space-focused stereotypes (Coleman et al., 2019). In this research, the statistically negative mediation effect and the insignificant direct effect suggested that people tend to avoid PLWHA’s living space when HIV-related information intrigues their emotional and cognitive reactions. For geographically segregated social groups, people might interfere that these groups were separated from the public because they were dangerous or threatening. This kind of perception raises the feeling of threat and negative evaluation for the surrounding environment, thus forming space-focused stereotypes for targeting social groups.

It was also worth noting that the mediation effect of threat perception was not as significant as community evaluation, demonstrated in the bootstrapped 95% *CI* ([−0.187, −0.008] versus [−0.370, −0.086]). There are several reasons for this result. First, the outcome variable of community approaching willingness is targeted to the community. Community is more strongly related to the cognitive mediator (i.e., community evaluation) than to the emotional mediator (i.e., threat perception); hence, it leads to a more prominent mediation effect of the community evaluation. Second, the measure of threat perception included the feelings of threat, fear, and anxiety but omitting disgust, which is more closely related to pathogen avoidance (Schaller and Park, 2011; Yang et al., 2020). The exclusion of disgust may have reduced the mediation effect of threat perception. Finally, apart from the threat, people also feel compassion when evaluating PLWHA (Kuah-Pearce and Guiheux, 2014). This research was mainly conducted with college students. They generally have a more liberal view of sexual behavior than older, more conservative generations (Yu, 2012). Many of the students had received HIV and AIDS education and had been shown to reduce bias towards PLWHA (e.g., Zha et al., 2021). Therefore, the feeling of compassion might moderate their feeling of threat in evaluating PLWHA’s living space. Further research could take all these factors into account for a more comprehensive approach to exploring the cognitive and emotional mechanism of people’s avoidance behavior regarding PLWHA.

## Limitations and Further Directions

This study is the first to examine space-focused stereotypes in Chinese culture and demonstrates the negative explicit space-focused stereotypes of PLWHA. It also discusses the influence of space-focused stereotypes on behavior and its mechanism, which have important theoretical and practical values. However, some limitations should be addressed in future studies.

One limitation concerns the target group. Although this study extended the space-focused stereotypes from Black Americans to PLWHA, it could not directly prove the universality of space-focused stereotypes. More importantly, the question remains as to whether there are direct connections between geographical segregation and space-focused stereotypes. Therefore, more geographically segregated social groups across cultures should be considered to explore the existence of space-focused stereotypes on these groups of people. Additionally, in light of the recent and ongoing COVID-19 pandemic, future research should continue to explore whether space-focused stereotypes can also be found in people infected with COVID-19, which is also segregated from the public. Related research should also help reveal the relationship between human cognition and societal change.

Another aspect lies in the explicit measures of space-focused stereotypes. When measuring the space-focused stereotype and community evaluation of PLWHA, we mainly constructed the items from the view of socio-economic status based on previous literature (Bonam et al., 2016; Yantis and Bonam, 2021). However, we could not simply conclude that space-focused stereotypes of PLWHA only exist in terms of economic-related community evaluations. There are other aspects to consider when evaluating PLWHA’s living spaces or communities. For example, whether PLWHA’s living spaces were also stereotyped as drug- or hospital-related (illustrated in Supplementary Table 1 in Supplementary Materials). Further research could extend the content of space-focused stereotypes and change the measurements according to the characteristics of the target social group.

Finally, for the manipulation of house information in Study 2, the HIV status could not be accessed when renting a house except the house owner decided to tell them. Thus, this manipulation lacked ecological validation. The present research is limited to negative evaluation and avoidance behavior for PLWHA related space. Future studies could apply a more ecologically valid approach in examining the influence of space-focused stereotypes of PLWHA. For example, as PLWHA are more likely to tell their intimate others about their HIV status, researchers could investigate the reactions of participants with differing levels of intimacy in relationship with PLWHA. The influence scope of PLWHA’s living spaces could also be an interesting research topic. Researchers could investigate the point at which participants would avoid living in a community altogether based on the number of PLWHA residents. Similarly, for a community with a certain number or percentage of PLWHA, investigators could examine the distance participants would consider acceptable to be able to live without worrying about infecting HIV?

## Conclusion

Two studies were conducted to investigate the existence and behavioral inclinations associated with space-focused stereotypes of PLWHA. The results demonstrated that space-focused stereotypes of PLWHA were explicitly significant, while the implicit GNAT test was not significant. Compared to those without HIV/AIDS, evaluation and behavioral inclinations toward the houses and living areas of PLWHA were more negative and manifested in community evaluation and community-approaching willingness. This research also revealed the emotional and cognitive pathways between space-focused stereotypes and behavioral inclinations, while threat perception and community evaluation mediated the effect of space-focused stereotypes on community-approaching willingness. Those who possessed a higher level of space-focused stereotypes of PLWHA were more inclined to avoid the houses and spaces of PLWHA. The findings from the present research suggest the existence of space-focused stereotypes with another geographically segregated social group, suggesting that cues related to space could also be a factor that forms stereotypes.

## Data Availability Statement

The raw data supporting the conclusions of this article will be made available by the authors, without undue reservation.

## Ethics Statement

The studies involving human participants were reviewed and approved by the Ethics Committee of the Center for Studies of Social Psychology. The patients/participants provided their written informed consent to participate in this study.

## Author Contributions

BZ and FW conceived and designed the whole experiment. YW and JY collected and analyzed the data. All authors contributed to the writing of this manuscript.

## Conflict of Interest

The authors declare that the research was conducted in the absence of any commercial or financial relationships that could be construed as a potential conflict of interest.

## Publisher’s Note

All claims expressed in this article are solely those of the authors and do not necessarily represent those of their affiliated organizations, or those of the publisher, the editors and the reviewers. Any product that may be evaluated in this article, or claim that may be made by its manufacturer, is not guaranteed or endorsed by the publisher.

## Footnotes

1
www.wjx.com
