# Supplementary material for: Space-Focused Stereotypes About People Living With HIV/AIDS and the Effects on Community-Approaching Willingness
Source: Front Psychol. 2022 Apr 15;13:772639. doi: 10.3389/fpsyg.2022.772639 (PMC9051341; doi:10.3389/fpsyg.2022.772639)
Supplement: Supplementary file 1 [file Table_1.docx]

**Table S.1** The full list of categories coded from participants’ space-focused stereotypes on people living with HIV/AIDS in Study 1A.

| Category | Characters | Number | Valence | | |  | Consensus | | |
| --- | --- | --- | --- | --- | --- | --- | --- | --- | --- |
|  |  |  | *M* (*SD*) | *t* | Cohen’s *d* | | *M* (*SD*) | *t* | Cohen’s *d* |
| Mess | chaos, clutter, disorder | 62 | -1.73(1.30) | -10.49^***^ | -1.33 | | 5.66(2.23) | 2.33^*^ | 0.30 |
| Dirty | unsanitary, unclean, dirty | 57 | -2.14(1.26) | -12.83^***^ | -1.70 | | 6.09(2.39) | 3.43^**^ | 0.45 |
| Dark | dim, dark, gloomy | 54 | -1.44(1.62) | -6.55^***^ | -0.89 | | 4.98(2.19) | -0.06 | -0.01 |
| Narrow | narrow, undersized, small | 52 | -1.19(1.07) | -8.06^***^ | -1.12 | | 5.46(1.91) | 1.74 | 0.24 |
| Chilly | white, empty, deserted | 38 | -0.84(0.89) | -5.86^***^ | -0.95 | | 5.53(1.84) | 1.76 | 0.29 |
| Isolation | isolated, quarantined | 38 | -1.84(1.13) | -10.07^***^ | -1.63 | | 6.50(2.11) | 4.37^***^ | 0.71 |
| Clean | clean, germfree, disinfection, sanitary | 35 | 1.77(1.09) | 9.64^***^ | 1.63 | | 5.40(2.32) | 1.02 | 0.17 |
| Remote | faraway, secluded, remote, converted | 30 | -0.90(1.40) | -3.53^***^ | -0.64 | | 6.50(2.03) | 4.05^***^ | 0.74 |
| Dilapidated | poverty, shabby, poor conditions | 29 | -1.52(1.24) | -6.57^***^ | -1.22 | | 6.03(1.66) | 3.36^**^ | 0.62 |
| Neat | organized, orderly, neat | 28 | 1.79(1.32) | 7.18^***^ | 1.36 | | 4.96(1.77) | -0.11 | -0.02 |
| Crowded | live with many other people, crowed | 27 | -1.07(1.14) | -4.89^***^ | -0.94 | | 5.56(1.19) | 2.43^*^ | 0.47 |
| Drug-related | lots of drugs, have needle tubing, smell of drugs | 23 | -0.61(1.67) | -1.75 | -0.36 | | 6.30(1.82) | 3.44^**^ | 0.72 |
| Ordinary | ordinary, common, normal | 17 | 0.82(0.95) | 3.57^**^ | 0.87 | | 5.76(2.46) | 1.28 | 0.31 |
| Quiet | quiet, silenced, oppressive | 16 | 0.13(1.45) | 0.34 | 0.09 | | 5.81(1.68) | 1.93 | 0.48 |
| Blocking | windows closed, stuffiness, enclosed | 14 | -1.71(1.33) | -4.84^***^ | -1.29 | | 5.71(2.13) | 1.26 | 0.34 |
| Bright | bright, sun-orientated | 14 | 2.07(1.14) | 6.79^***^ | 1.82 | | 5.00(2.51) | 0.00 | 0.00 |
| Spacious | spacious, wide, roomy | 12 | 1.42(1.31) | 3.74^**^ | 1.08 | | 4.50(1.88) | -0.92 | -0.27 |
| Dangerous | dangerous, poor public security | 11 | -2.73(0.65) | -13.99^***^ | -4.22 | | 5.82(3.12) | 0.87 | 0.26 |
| Simple | simple, concise, austerity | 11 | 0.18(0.60) | 1.00 | 0.30 | | 6.36(1.21) | 3.75^**^ | 1.13 |
| Comfort | comfort, soft | 10 | 2.30(0.82) | 8.83^***^ | 2.79 | | 5.10(2.18) | 0.14 | 0.05 |
| Noisy | noisy, bustle, downtown | 9 | -1.50(1.18) | -4.02^**^ | -1.27 | | 5.30(2.16) | 0.44 | 0.14 |
| Depressed | feel of tension, depressed, pressure in the air | 8 | -1.78(0.83) | -6.40^***^ | -2.13 | | 5.56(1.33) | 1.25 | 0.42 |
| Warmth | warmth | 5 | 2.00(0.71) | 6.33^**^ | 2.83 | | 3.60(1.95) | -1.61 | -0.72 |
| Safety | safety, social-secure | 4 | 1.75(1.26) | 2.782 | 1.39 | | 3.75(1.89) | -1.32 | -0.66 |
| Lively | prosperous, lively | 3 | 2.00 |  | | | 4.00 |  | |
| Drying | drying | 2 | 0.00 |  | | | 4.50 |  | |
