# Supplementary material for: Space-Focused Stereotypes About People Living With HIV/AIDS and the Effects on Community-Approaching Willingness
Source: Front Psychol. 2022 Apr 15;13:772639. doi: 10.3389/fpsyg.2022.772639 (PMC9051341; doi:10.3389/fpsyg.2022.772639)
Supplement: Supplementary file 2 [file Table_2.docx]

**Table S.2** The full list of positive and negative attributes used in GNAT.

| Positive attributes | | | |  | Negative attributes | | | |
| --- | --- | --- | --- | --- | --- | --- | --- | --- |
| Attributes | *M* | *SD* | *t* |  | Attributes | *M* | *SD* | *t* |
| Formal trials | | | | | | | | |
| Advanced | 6.07 | 1.04 | 16.90^***^ |  | Unclean | 2.07 | 1.08 | -15.18^***^ |
| In order | 5.94 | 1.01 | 16.41^***^ |  | Mess | 2.33 | 1.05 | -13.49^***^ |
| Neat | 5.92 | 1.10 | 14.82^***^ |  | Dangerous | 2.24 | 1.26 | -11.87^***^ |
| Safety | 5.96 | 1.17 | 14.23^***^ |  | Shabby | 2.53 | 1.18 | -11.30^***^ |
| Clean | 5.86 | 1.13 | 13.98^***^ |  | Disorder | 2.40 | 1.26 | -10.73^***^ |
| Comfort | 5.93 | 1.20 | 13.62^***^ |  | Poor | 2.53 | 1.18 | -10.64^***^ |
| Renewed | 5.76 | 1.11 | 13.52^***^ |  | Remote | 2.76 | 1.00 | -10.49^***^ |
| Wealthy | 5.50 | 1.13 | 11.31^***^ |  | Crowded | 2.74 | 1.11 | -9.63^***^ |
| Practice trials | | | | | | | | |
| Spacious | 5.38 | 1.17 | 9.99^***^ |  | Narrow | 2.83 | 1.16 | -8.51^***^ |
| Lively | 5.04 | 1.40 | 6.32^***^ |  | Chill | 3.31 | 1.15 | -5.14^***^ |

^***^ *p* < .001.
