# Supplementary material for: Space-Focused Stereotypes About People Living With HIV/AIDS and the Effects on Community-Approaching Willingness
Source: Front Psychol. 2022 Apr 15;13:772639. doi: 10.3389/fpsyg.2022.772639 (PMC9051341; doi:10.3389/fpsyg.2022.772639)
Supplement: Supplementary file 3 [file Table_3.docx]

**Table S.3** The original Chinese version of categories in **Table S.1** and positive attributes in **Table S.2**.

| Categories | 类别 |  | Categories | 类别 |
| --- | --- | --- | --- | --- |
| Table S.1 |  |  |  |  |
| Mess | 杂乱 |  | Quiet | 安静 |
| Dirty | 脏 |  | Blocking | 封闭 |
| Dark | 阴暗 |  | Bright | 明亮 |
| Narrow | 狭小 |  | Spacious | 宽敞 |
| Chilly | 清冷 |  | Dangerous | 危险 |
| Isolation | 孤单 |  | Simple | 简单 |
| Clean | 干净 |  | Comfort | 舒适 |
| Remote | 偏僻 |  | Noisy | 嘈杂 |
| Dilapidated | 破旧 |  | Depressed | 压抑 |
| Neat | 整齐 |  | Warmth | 温暖 |
| Crowded | 拥挤 |  | Safety | 安全 |
| Drug-related | 药物 |  | Lively | 热闹 |
| Ordinary | 普通 |  | Drying | 干燥 |
| Table S.2 | | | | |
| Advanced | 先进的 |  | Comfort | 舒适 |
| In order | 有条理 |  | Renewed | 崭新 |
| Neat | 整洁 |  | Wealthy | 富有 |
| Safety | 安全 |  | Spacious | 宽敞 |
| Clean | 干净 |  | Lively | 热闹 |
